# Discrimination between Fresh and Frozen-Thawed Fish Involved in Food Safety and Fraud Protection

**DOI:** 10.3390/foods9121896

**Published:** 2020-12-18

**Authors:** Luca Maria Chiesa, Radmila Pavlovic, Maria Nobile, Federica Di Cesare, Renato Malandra, Davide Pessina, Sara Panseri

**Affiliations:** 1 Department of Health, Animal Science and Food Safety, Università Degli Studi di Milano, Via Celoria 10, 20133 Milan, Italy; luca.chiesa@unimi.it (L.M.C.); maria.nobile1@unimi.it (M.N.); federica.dicesare@unimi.it (F.D.C.); sara.panseri@unimi.it (S.P.); 2 ATS Milano-Città Metropolitana, Director of Veterinary Unit, 20122 Milano, Italy; rmelandra@asl.milano.it; 3 Quality Department, Italian Retail Il Gigante SpA, 20133 Milan, Italy; pessina@ilgigante.net

**Keywords:** food safety, freezing, thawing, high resolution mass spectrometry, Atlantic salmon, bullet tuna, amino acids, metabolomics, fish

## Abstract

This study aims to discriminate fresh fish from frozen/thawed by identification of the key metabolites that are altered during the freezing/thawing processing. Atlantic salmon (*Salmo salar)* and bullet tuna (*Auxis rochei*) were selected as they are representative of broad consumption, and susceptible to pathogen contamination. Atlantic salmon samples were subjected to the following regimes: −20 °C (24h) and −35 °C (15 h) freezing, then thawed respectively in the blast chiller and in the cold room and analyzed immediately or after 10 days; (2) bullet tuna samples were frozen at −18 °C and thawed after 15, 30 and 90 days. High resolution mass spectrometry based on untargeted metabolomic analyses and statistical data treatment confirmed significant variations in the quantity of certain metabolites: the amount of l-phenylalanine in salmon increased immediately after thawing while that of anserine decreased. The concentration of l-arginine and its metabolites was altered at the 10th day after thawing rendering them promising markers of salmon freezing/thawing. As regards bullet tuna, compounds resulting from lipid degradation (l-α-Glyceryl-phosphoryl-choline and N-methyl-ethanolamine phosphate) increased notably during the storage period. This approach could be used to reveal common fraudulent incidents such as deliberate replacement of fresh fish with frozen/thawed, with food safety risks as the primary implication.

## 1. Introduction

In the fish industry, the term “fresh” denotes that the fish has never been frozen, starting from its capture until its commercialization. Fresh fish is an extremely delicate food, susceptible to spoilage during fishery management, processing, transporting, packaging, and storage. Quality declines quickly after harvesting, requiring instantaneous adequate processing in order to preserve nutritional properties and avoid safety hazards. Freezing is recognized as an efficient method for safeguarding fish. Although it is feasible to obtain frozen fish of a high nutritional quality, the quality of fresh fish is deemed superior [1]. The preferences of consumers for fresh fish are based on sensory characterization, as apparent alterations in the flavor, odor, consistency and color of flesh can occur during freezing, frozen storage and thawing [2].

Nevertheless, some producers or distributors may attempt to sell frozen/thawed fish declaring it to be fresh in order to increase profits. This practice is a quite common form of deceit in fish commercialization. Generally, food safety in the aquaculture sector has been intensified, due to rapid action undertaken in global fish and fish products management. Fraud, defined as “the intentional act of substituting, adding, adulterating, tampering, or misrepresentation of ingredients and/or packaging” [3], represents a primary threat to seafood production and commercialization, especially regarding species that are widely consumed.

Salmon and tuna species are among the few types of fish preferred by consumers. Both are widely appreciated: high-quality proteins, essential fatty acids content, substantial amounts of minerals and vitamins make them popular among seafood eaters. The worldwide production of Atlantic salmon (*Salmo salar*) increased by around 7% in 2019, to just over 2.6 million tons, while predictions for 2020 provided for an additional 4–5% [4]. About 70% of the world’s salmon production is farmed, and most of this comes from Norway, Chile, Scotland, and Canada [5]. Bullet tuna is an important high yield fish, with a continuing growth in annual production, more than 120,000 tons/year [6]. Bullet tuna is moderately priced valuable commercially important commodity which has characteristics akin to mackerel and tuna. Apart its availability as fresh fish, the production and distribution of frozen bullet tuna filets has become popular in recent years.

Fresh fish is vulnerable to attack by parasites, among which *Anisakis simplex* is the most common [7]. This might represent a hazard to consumer health if the fish is consumed raw, marinated or partially cooked. Conversely, both cooking and freezing kill parasites and thus guarantee the safety of fish products as regards this risk. To better protect the health of consumers, European legislation has obliged food business operators to subject all fishery products to be consumed raw to heat declination [8]. This regulation is based on European Food Safety Authority (EFSA) scientific opinion [9], which claims that “freezing treatments remain the most effective processes guaranteeing the killing of parasitic larvae under well-defined conditions”. Treatment that provides reliable protection is freezing at −20 °C for not less than 24 h or at −35 °C for at least 15 h. In the same document [9] it was emphasized that if Atlantic salmon is farmed and fed according to the strict instructions, the probability of *Anisakis simplex* infection is minimal. Regrettably, sufficient monitoring data that would identify which species present a health hazard as regards the presence of parasites were not available for any other farmed fish.

Details concerning the storage technique applied in fish post-harvest maintenance denote one of the strategic issues aimed at guaranteeing consumer safety. Regulations EU No 1169/2011 [10] and No 1379/2013 [11] established that the physical condition of the seafood and preservation treatment applied must be labelled. In the case of a food product that has been frozen and then sold thawed, the name of the food shall be supplemented by the term ‘defrosted’.

As exchanging frozen/thawed for fresh seafood is a fraudulent practice, this requires the introduction of robust analytical procedures for freshness evaluation. The methods that have been used to distinguish fresh from frozen/thawed could be categorized into the following categories: organoleptic, histological, enzymatic, physical, physiological and biochemical. The literature survey of the most representative analytical approaches is presented in Table 1.

Organoleptic methodology may not give a reliable answer to questions regarding thermal processing [16] as coosing and evaluating individuals who conduct the testing is quite subjective. As a matter of fact, it is very difficult to distinguish fresh from frozen-thawed fish considering only the organoleptic characteristics [25,26].

Histological methods are very precise and are based on highlighting the rupture of muscle tissue and on the presence of voids in muscle cells, which verify the low-temperature management of fish [3,12,13,14]. However, in production and commercial conditions, this method is not easy to perform; it requires special training of personnel and laboratory equipment. Moreover, histological examination requires a long time, which might be challenging in the conditions of real circulation of samples to be analyzed [15].

Enzymatic methods reveal alterations in the activity of different mitochondrial enzymes and are based on the fact that during thawing, the water-enriched external medium increases hydrostatic pressure in cells and induces the rupture of plasma membrane, provoking an enrichment of intracellular enzymes in the final exudate [24]. The main drawback of this approach is the high variation in fresh samples, which could actually diminish the variances between fresh and frozen-thawed samples. A physical approach relies on determination of the dielectric properties of the tissues, which is considered less specific. Physiological methods measure the changes in the fish eye lenses, hematocrit profile, or detect alternations in the fluorescence of fish muscle [15,22,25].

Biochemical analysis, supported by chemometric techniques has been emerging as a potent tool in food fraud analytics [27], as it combines multivariate data analysis with high-throughput instrumental techniques such high pressure liquid chromatography coupled with high-resolution mass spectrometry (HPLC-HRMS) or nuclear magnetic resonance (NMR). Particularly, NMR has been proven to be able to detect metabolic alterations caused by different kinds of fish processing [28].

Present-day research that regards the alterations of the main metabolic pathways in fish flesh caused by freezing/defrosting discovered by a metabolomic approach is limited. NMR instrumental evaluation has been performed just recently [21] while HPLC-HRMS, to the best of our knowledge, has not yet been applied. Thus, the dynamic changes in metabolic components and the mechanisms underlying fish meat thermal manipulation during frozen storage and subsequent thawing remain unclear. Freezing and thawing handlings can considerably affect the safety and quality of frozen fish, particularly in terms of the cellular structure and protein/free amino acid pool [29].

In view of these concerns, the main objective of this study was to identify the key metabolites that are altered during freeze/thaw processing by using HRMS based untargeted metabolomics as well as to investigate the feasibility of this methodology to provide more careful inspective controls in two specific cases: (1) Atlantic salmon since it is a fish species that is widely sold as fresh and intended to be consumed raw and (2) bullet tuna that is commercialized both fresh and frozen.

## 2. Materials and Methods

### 2.1. Chemicals and Reagents

Standard mix of 22 amino acids, biogenic amines (histamine, putrescine, cadaverine, spermidine, spermine), creatine, creatinine, anserine, hypoxanthine, xanthine and internal standard (IS, 1,7-diaminoheptane) heptafluorobutyric acid (>99%) were obtained from (Sigma-Aldrich, MerckKGaA, Darmstadt, Germany) while acetonitrile and perchloric acid (70–72%) were obtained from VWR (Darmstadt, Germany). Milli-Qsystem (Millipore, Merck KGaA, Darmstadt, Germany) was used for water purification.

### 2.2. Experimental Design

#### 2.2.1. Atlantic salmon (*Salmo salar*) Rapid Freezing/Thawing Processing

**Figure 1:**
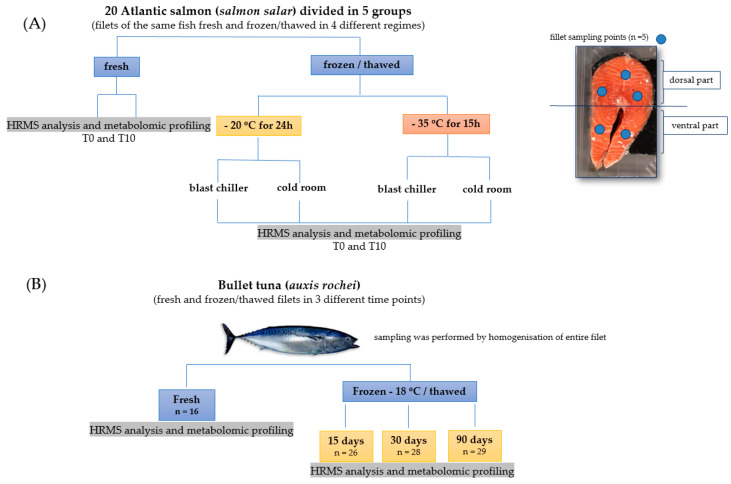
Experimental design adopted in this research for Atlantic salmon (**A**) and bullet tuna (**B**).

Five fillets were taken, from each one of 20 fresh Atlantic salmon samples, one for the fresh and four for the categories subjected to freezing/thawing treatments presented in Figure 1A. The samples were frozen at two different temperatures: −20 °C and −35 °C and defrosted in two regimes in a blast chiller and in a cold room, selected as conditions suitable to prevent health risks. All the samples, both fresh and exposed to four different freezing/thawing modes, were subjected to analysis: (1) at day 0, immediately after defrosting (T0) and (2) after 10 days (T10) of conservation under a refrigeration regime (0–2 °C).

#### 2.2.2. Bullet Tuna (*Auxis rochei*) Long Term Freezing

Fresh filets of the same batch of bullet tuna were chosen randomly and divided into 4 groups (Figure 1B). The fresh ones (16) were analyzed immediately upon arrival, while the others were frozen in a freezer at −18 °C for 15 days (26 pieces), 30 days (28 pieces) and 90 days (29 filets) and analyzed after defrosting at room temperature.

### 2.3. Sample Preparation

The samples were managed according to the method published for the determination of biogenic amines [30] with slight modifications. A homogenized sample (0.8 g) was placed in a 50 mL centrifuge tube and 40 mL of perchloric acid (0.1 mol/L) was added. After vortex homogenization for 2 min, centrifugation at 5000 rpm for 10 min (4 °C) was performed. Clear extract (2 mL) of was filtered into a test tube from which 100 µL was transferred into the HPLC vial, spiked with 10 µL of internal standard IS (10 and µg g^−1^) with the addition of 890 µL of the initial HPLC mobile phase. 10 µL of the resultant mixture was injected.

### 2.4. HPLC Q-Exactive Orbitrap HRMS Analysis

In order to perform HPLC-Q-Exactive-Orbitrap^®^-MS analysis, chromatography was accomplished on an HPLC Surveyor MS platform (Thermo Fisher Scientific, San Jose, CA, USA) using a Synergi Hydro RP column (150 × 2 mm i.d., 4 μm, (4 × 3 mm i.d.) (Phenomenex, Torrance, CA, USA) as stationary phase. The mobile phase involved water (A) and methanol (B) both acidified with 0.05% heptafluorobutyric acid. The gradient (0.3 mL/min flow rate), begun with 98% of eluent A with a linear reduction to 50% in 5 min, that was constant in the next 3 min. The initial conditions returned at 8 min, tracked by a 5-min re-equilibration period. The column department temperature was set at 30 °C and while autosampler was kept at 5 °C.

Q-Exactive Plus HRMS (Thermo Scientific, San Jose, CA, USA) operated in positive mode with following operative parameters of heated electrospray ionisation (HESI) source: 3.4 kV for capillary voltage, 290 °C for capillary temperature and 280 °C for auxiliary gas heater temperature. Sheath and auxiliary gas were adjusted at 35 and 15 arbitrary units, while S lens RF level was set at 55. The full scan (FS) with resolving power 140,000 (scan range of *m/z* 70–400) was used for the screening and statistical evaluation of the chromatographic profiles. Full scan data-dependent acquisition (FS-dd-MS^2^) with resolving power 70.000 and 17.500 for FS and dd-MS^2^, respectively, was employed for fragmentation of pseudo-molecular ions detected in FS mode. The automatic gain control (AGC) in FS acquisition mode was set at 3 × 10^6^, with an injection time of 200 ms, while for FS-dd-MS^2^ the AGC target was 2 × 10^5^, with the auto-regulated injection time. Fragmentation of precursors was executed with stepped, normalized collision energy (NCE) set at 20 eV and 30 eV.

To confirm a satisfactory permanence of sequence analysis, all biological samples were run in duplicate along with quality controls (QC) that were employed randomly through the analytical batch. QC samples were prepared by pooling the same volume from the real samples for each experiment. In addition, a procedural blank sample was involved in each batch to identify an eliminate the background signals.

### 2.5. Untargeted Metabolomics with Compound Discoverer™ Workflow

The Q Exactive Orbitrap raw data obtained either from FS or from FS-dd-MS^2^ were analyzed by Compound Discover 3.0 software (Thermo Fisher, MA, USA) that enabled programmed peak identification. The procedure is based on a series of steps that are accomplished consequentially as presented in the workflow diagram (Supplementary Materials Figure S1). Input files were labeled appropriately: procedural blank, QC and sample type (fresh and different methods of freezing/thawing). The first step consists of spectra selection, and was followed by “alignment of retention time” processing that was based on an adaptive curve with a maximum retention time shift set at 0.25 min. The third block was to select the spectra and to identify precursor ions with maximum mass tolerance of 2 ppm. The “group compound” block involved categorization of detected compounds according to the engaged databases *m*/*z* Cloud: ChemSpider ((https://www.chemspider.com) and *m*/*z* Cloud). The “fill gaps” step is a key phase for automatic adjustment of detected signals, for bypassing error interpretation due to eventual absence of the peaks in some samples or the presence of certain signals as background compounds in blank samples. Finally, in order to obtain more uniform signals, data were normalized by constant sum model that was followed by assignation of background compounds.

Criteria for putative identification of metabolites identified by the above-described Compound Discoverer workflow were chosen as a combination of several different assets: an mzCloud match score higher than 80% and the same identification being proposed by at least one external web database: Human Metabolome platform HMDB (https://hmdb.ca/), Kyoto Encyclopedia of Genes and Genomes (KEGG), (https://www.genome.jp/kegg), Pubchem (www.pubchem.com) or Small Molecule Pathway Database (SMPDB) (http://smpdb.ca). If the mass fragmentation pattern did not correspond to any of the databases from Compound Discoverer™ software (Thermo Fisher, MA, USA) manual verification of the fragmentation pattern program was performed. Authentic chemical standards of amino acids, biogenic amines and nucleotides were used to confirm putative identification.

Differential analysis, as a part of Compound Discoverer workflow, involved principal component analysis (PCA) and volcano plot (VP) processing. PCA was based on the first two principal variables while VPs were created on Log_2_ (FC) and −log_10_ (*p*-value) used to filter metabolites of interest and to identify the main differentiators. Moreover, box-and-whiskers plots with descriptive statistics for chosen compounds were used.

Semi-quantification was performed using the internal standard method and the final results were presented as IS equivalents (µg/g). Differences between groups for semi-quantitative analysis were identified using a one-way ANOVA followed by Dunnett post hoc test from the BioVinci statistical program (Version 1.1.4., BioTurning Inc., San Diego, CA, USA, 2018). A *p*-value of less than 0.05 was considered statistically significant.

## 3. Results and Discussion

Metabolomics in general have been employed to monitor real time, dynamic changes in metabolites in different fish and fish products during storage and throughout various processing conditions [28,29]. This methodology offers an essential “omics” concept to elucidate the principal mechanisms underlying modifications in food quality and to provide solutions that would improve food preservation techniques. Nevertheless, HRMS based metabolomics has not yet been fully exploited for fish matrices, although high performance liquid chromatography-benchtop quadrupole Orbitrap hybrid mass spectrometry has been used for multiresidual analysis in fish and seafood products [31,32]. Therefore, as the main goal of this research was to detect reliable metabolites fingerprints that change during freezing/thawing processing of fish meat it was necessary, firstly, to establish an adequate experimental design along with a suitable HRMS/Compound Discoverer instrumental workflow.

### 3.1. HRMS Untargeted Metabolomics with Compound Discoverer™ Workflow

**Figure 2:**
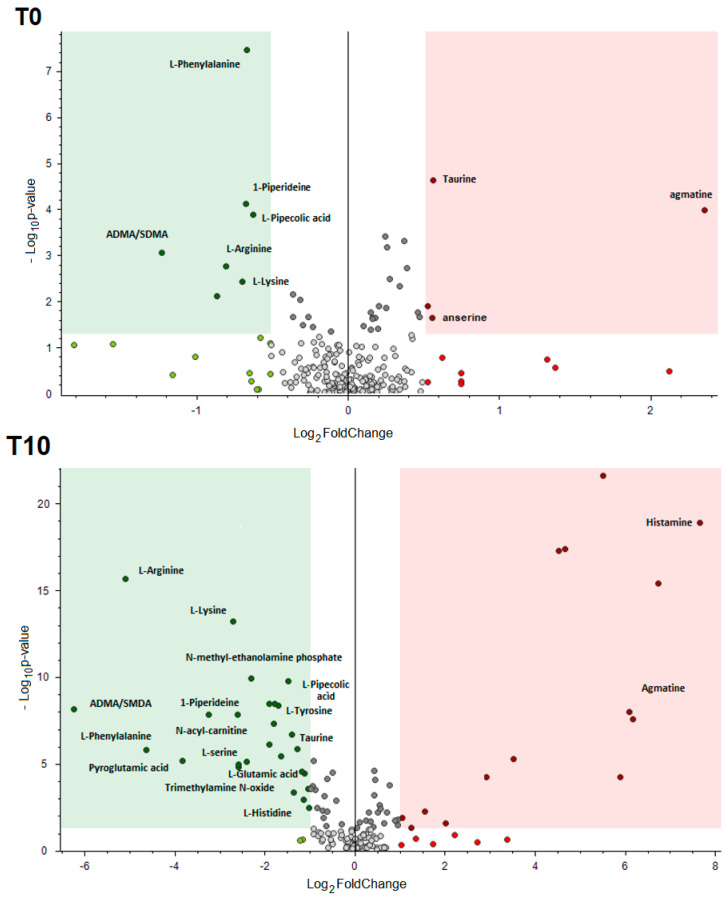
Volcano plot comparison between the relative intensity of chromatographic peak from fresh and pulled frozen/thawed salmon samples. The green region contains up-regulated signals with intensities from fresh samples significantly lower than those from pulled frozen/thawed and were greater than the upper fold-change (FC) threshold. The red region includes down-regulated peaks where the intensities from fresh were significantly higher than those from pulled frozen/thawed and was less than the lower FC threshold. *p*-value (PV) = 0.05.

**Figure 3:**
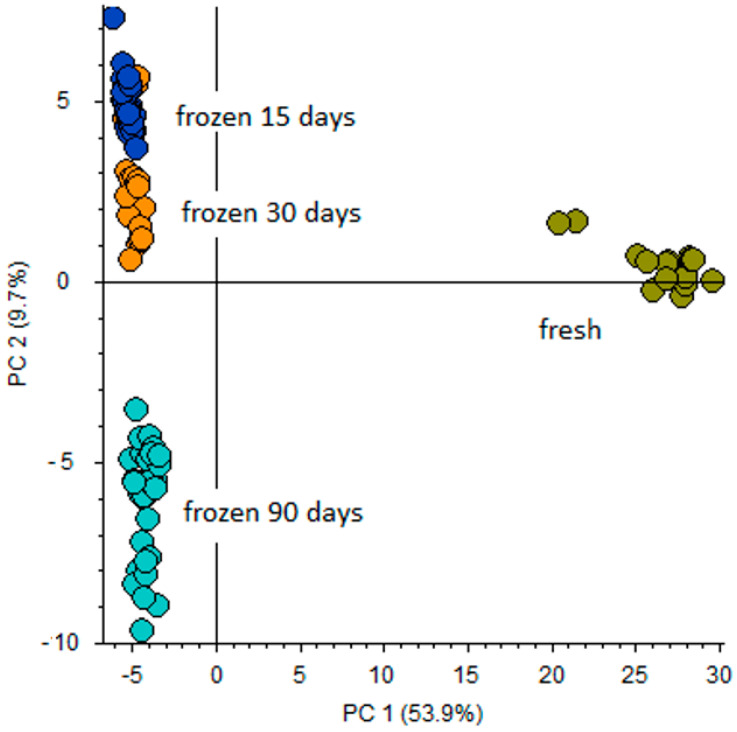
Principal component analysis (PCA) projection on the distribution of fresh and pulled frozen/thawed bullet tuna samples as regards PC-1 with PC-2 when high resolution mass spectrometry (HRMS) spectral characterization of normalized peak area was used.

In order to perform untargeted profiling of fresh and frozen/thawed fish with subsequent data processing performed by Compound Discoverer™ (CD) software, two types of Q-Exactive-Orbitrap acquisition mode were implemented:

1. **Full scan (FS)** with resolving power of 140,000 identified signals of metabolites; 156 and 148 candidates for Atlantic salmon and bullet tuna, respectively. Applying the Compound Discoverer workflow described above, differential analysis that involved principal component analysis (PCA) and volcano plot (VP) was performed. PCA was used in order to visualize and provide overall differentiation between fresh and frozen/thawed samples. In order to obtain more uniform signals, data were normalized by conversion of the absolute peak areas for each metabolic feature into relative peak intensities. This data was applied to PCA and VP evaluation in order to visualize maximum variation. As preliminary statistical assessment did not give any differences between the freezing regimens used for Atlantic salmon and for bullet tuna, respectively, both PCA and VP were performed comparing the fresh samples to the group of frozen ones, regardless of the freezing/thawing treatment applied. PCA (Figure S2) clearly distinguished the fresh and frozen/thawed sample at T10 for salmon samples, while at T0 there were no differences. Furthermore, VP (Figure 2) analysis gave more precise responses regarding the main differentiators. For the bullet tuna, the PCA score plot clearly distinguished the fresh filets from frozen ones, with notable differences between 90-day samples and the two shorter sampling periods (Figure 3).
2. **FS-data dependent (FS-DDA)** processing was performed on the selected inclusion list of 156 and 148 signals extracted from the FS data collection. This acquisition mode allowed putative identification of the main differentiators and was based on predicted composition according to accurate mass, adduct formation, isotopes ratios and fragmentation patterns. Selection of the best-fit candidates for the signals was performed by software-linked mass fragmentation database libraries (mzCloud and ChemSpider). In some cases, as no satisfactory confirmation was found from existing databases (including external libraries like Human Metabolome Database) presumption of the structural formula was achieved in-house by identification of fragment structure.

### 3.2. Salmon Metabolite Profile upon Rapid Freezing/Thawing Processing

European Union regulation 1276/2011 [8] requires that fishery products intended for raw consumption be frozen at −20 °C for not less than 24 h or −35 °C for at least 15 h to prevent disease caused by various parasites. From a metabolomic viewpoint, the impact of such obligatory treatment on small polar molecule profile and composition has not yet been exploited. It is well established that many protein conformational and configurational alterations accompanied with elementary composition deviations occur during freeze storage of salmon [1]. The influence of freezing on fish muscle proteins mainly concerns changes in the solubility of protein fractions, their water-holding capacity, and the activity of proteolytic enzymes [24]. Freezing causes disruption and separation of muscle fibre bundles due to the occurrence of numerous extracellular and intracellular ice crystals. Since during freezing muscles undergo changes that include formation of ice crystals and rupture of cell membranes, upon thawing water with soluble components is released from the muscle tissues [1].

Using the Compound Discoverer platform described above, the presence of 36 compounds divided into categories was confirmed for the Atlantic salmon cold chill experiment. Apart from the Compound Discoverer workflow that included differential analysis, semi-quantitative evaluation was accomplished using the internal standard procedure and the results were finally expressed as µg/g IS equivalents. The metabolites that varied significantly due to freezing treatment are presented in Table 2, while all metabolites identified are given in Tables S1 and S2.

The compounds revealed belong predominately to the free amino acids/small peptides pool, but some important nucleotides were also detected. Basically, there were no differences between treatments, and data were processed in order to compare the fresh samples with pooled fresh/thawed by volcano plot evaluation (Figure 2). It can be noted that there were just few metabolites with significant alterations in T0, while the storage period of 10 days after defrosting of samples revealed more metabolites. Another important finding is the elevated concentration of phenylalanine only at T0, which is accordance with newly published findings [21].

The most abundant metabolites were creatine and anserine, and this is in line with recent research [29]. During refrigeration, the integrity of fish flesh proteins is compromised and their decomposition could produce a variety of intermediary metabolites, first of all small molecular peptides and free amino acids. Anserine (β-alanyl-N-methyl histidine) is a histidine dipeptide that is found in high concentrations in the skeletal muscles of fish. It known as a scavenger of reactive oxygen species and acts as a pH regulator (especially in acidic conditions) due to the presence of an imidazole heterocyclic structure [33]. The fact that a significant decrease of anserine was found in the frozen/thawed sample at T0 candidates this dipeptide as a promising marker for detection of this treatment immediately after thawing.

Tables S1 and S2 as well as Figure S3 show the most important amino acids whose concentration was significantly different especially 10 days after the thawing of frozen samples. Many free amino acids are closely related to fish flavor and can be used as indicators of quality and freshness [34], including arginine, serine, and methionine. The alterations in the amino acids pool is most probably due to the actions of endogenous proteases and microorganisms that enhance free amino acid generation as was demonstrated for chicken meat [35].

The most important finding of this study is the modification in the l-arginine metabolic pathway. l-arginine increased at both time points, with concomitant agmatine reduction and an increase in methylated l-arginine. This indication of increased enzymatic activity of some protein arginine methyltransferases (PRMTs) that play a major role in the methylation of proteins having an arginine residue, catalyzing both the asymmetric dimethylation (ADMA) and symmetric dimethylation of arginine (SDMA) PRMTs has not yet been studied extensively in fish [36], and therefore, deserves further elucidation. Moreover, the l-arginine metabolic pathway evaluated by our metabolomic strategy revealed a substantial decline in agmatine concentration. This result is quite surprising as agmatine belongs to the class of biogenic amines commonly related to fish spoilage, since they accumulate as a result of an on-going proteolysis and amino acid decarboxylase activity of microorganisms. Fish freshness has been evaluated using more than one single biogenic amine [37]. In the experimental design of this study, the utilization of biogenic amines as indicators was not relevant because of the low accumulation rate detected (Tables S1 and S2). The reason why a notable decrease in agmatine concentration occurs remains to be clarified.

### 3.3. Bullet Tuna Metabolites Profile upon Long Freezing/Thawing Processing

Fish meat of tuna and salmon possess different characteristics predominantly caused by the fact that tuna is captured wild in tropical waters and salmon is farmed in colder waters. Nevertheless, no prominent variances were detected in the amino acid profiles of either species except the higher histidine concentration reported for tuna [29]. This finding is also confirmed by our results (Table S3). High endogenous histidine concentration should be taken into consideration very carefully, as some bacteria can decarboxylate free histidine to histamine, which can cause a disorder named scombroid fish poisoning. In this study, in spite of the high histidine concentration, histamine production in bullet tuna was non-detectable (Table S3). It is worth noting that high concentration 3-methyl-histidine was detected and its decarboxylated counterpart 3-methyl-histidine was present, as well.

During a prolonged freezing period, the concentration of almost all metabolites decreased notably (Table S3) most probably due to the linkage of ice crystals to muscle, which triggered a pronounced dilution status of bullet tuna muscle samples upon defrosting. This provoked a clear metabolomic distinction between fresh and frozen/thawed samples (Figure 3). Results obtained regarding the semi-quantification of amino acids and their metabolites show that variation in freezing storage time did not change the relative quantities of metabolites, but confirmed the finding of Reis et al. [18] regarding the water content managed through experimental design and modelling. This is a crucial point to be taken into account, especially when commercial samples are to be analyzed and where variables such as bounded water and/or freezing temperature are difficult to control.

Another hypothesis would be that as freezing is never an instantaneous process, metabolomic alternations in external fillet part could be different from those that are going on in internal fillet portion where freezing process is prolonged. The ice crystal that are firstly formed on the surface does not preclude water under ice being a liquid for a while, thus enzyme kinetics might not be uniform in all frozen fillet parts, and this is influenced by storage time.

l-arginine did not behave as all other amino acids (Table 3). Its concentration continued to increase, as happened with salmon species, which candidates this semi-essential amino acid as a universal marker of fish freezing/thawing processing. In contrast to the salmon metabolite profile, few metabolites emerged as specific for this fish species (Table 3). Particularly, γ-glutamyl-S-methylcysteinyl-β-alanine, known as S-methylhomoglutathione, which is involved in the glutathione metabolic pathway, has not been reported for fish so far.

Nevertheless, the most important finding of this experiment is an increase in the concentration of two water-soluble phospholipid metabolites: l-α-glyceryl-phosphoryl-choline and N-methyl-ethanolamine phosphate upon thawing of frozen samples regardless of storage period. Total lipid content generally decreases in fish flesh during cold storage [38], because triglycerides and phospholipids are hydrolyzed by lipolytic enzymes such as lipase and phospholipase A2 and are additionally susceptible to oxidation [39]. However, reductions in total lipid content vary notably from fish to fish species, depending especially on phospholipids fraction. Literature data for phospholipid composition of bullet tuna is rare, but increases in l-α-glyceryl-phosphoryl-choline and N-methyl-ethanolamine-phosphate in the frozen/thawed samples in comparison to fresh point towards accentuated phospholipid catabolism and lipid oxidation that is, as reported recently, induced by pro-oxidative action of reduced myoglobin [40]. As a matter of fact, bullet tuna muscle is rich in myoglobin and phospholipids with unsaturated fatty acids [41]. Consequently, the discoloration caused by formation of meth-myoglobin and lipid oxidation can easily take place during freeze storage, which remarkably encumbrances the processing and utilization of this species.

The accumulation of water-soluble phosphodiesters in perchloric extracts of diverse fish tissues caused by physiological/osmotic stress was demonstrated with remarkable interspecies qualitative and quantitative differences [42]. Therefore, it is reasonable to speculate that the higher levels of two cytosoluble phosphodiesters, observed in our frozen bullet tuna samples are provoked by impaired phospholipid membrane integrity due to mechanical damage caused by ice crystal formation. Additionally, here the ice formation and its distribution is complex process: due to chrioscopic effect of water soluble compounds a certain delay occurs between getting a nominal freezing temperature and the time required for that same temperature to reach the center of a fish fillet.

Taken together, our metabolomic profiling for bullet tuna highlighted three metabolites as freezing markers: l-arginine, l-α-glyceryl-phosphoryl-choline and N-methyl-ethanolamine (Table 3).

## 4. Conclusions

In conclusion, this study presents results on the metabolomic profiling of Atlantic salmon and bullet tuna, two frequently consumed fish species that are usually stored in a different manner, according to final consumption modality. Some promising indicators were identified: arginine and its metabolites in Atlantic salmon and phosphated choline/etanolamine derivates in particular for bullet tuna. Further studies on the effect of applied freezing/thawing regimes are mandatory in order to establish a controlled food safety model system that, accompanied by eventual evaluation of enzymes involved in the formation of the above-mentioned metabolites, would be able to clearly distinguish between fresh and freezing/thawing management. However, our fast and accurate qualitative metabolomic investigation defined the specific indicators of freezing/thawing processing concerning both short and prolonged storage time periods that can be readily applied as a reliable tool in order to support industry for the rapid identification of potential fraudulent labelling of frozen-thawed fish.

## Figures and Tables

**Table 1:** Literature data regarding the studies that differentiate fresh from frozen/thawed fish.

Reference	Fish Species	Method Applied for Differentiation	Identification Criteria/Markers Suggested
Histological methodology			
Bozzetta et al., 2012 [12]	Gilthead, red mullet, swordfish	Specific microscopic changes related to freezing transverse muscle sections with hematoxylin and eosin staining	- microscopic characteristics of the muscle fibers - vacuoles of various dimensions, optically empty or filled with eosinophilic material caused by ice crystals in frozen samples
Meistro et al., 2016 [13]	Marinated anchovy		
Pezzolato et al., 2020 [3]	Smoked salmon		
Tinacci et al., 2018 [14]	European hake	Standard histological morphological and morphometric parameters	- structural score - presence of freezing vacuoles - vacuoles amount - presence of interstitial material
Orlova et al., 2020 [15]	Not specified	Hematoxylin-eosin staining and microscopy	presence of fibrous areas, tearing of muscle fibers
Organoleptic evaluation			
Watanabe et al., 2020 [16]	Sashimi from pacific saury	Consumer panel sensorial evaluation	taste and flavor, fattiness, smooth mouth feel, retro-nasal aroma, redness of meat, overall appearance, texture, springy palatability, wateriness
Spectroscopy-based approach			
Kimiya et al., 2013 [17]	Salmon	Visible-Near InfraRed Spectroscopy (Vis-NIRS)	spectral changes due to oxidation of heme proteins
Reis et al., 2017 [18]	Tuna		chemometrics based on structural alternations of myoglobin and its oxidative products
Solid phase microextraction gas chromatography/mass spectrometry (SPME-GC/MS)			
Leduc et al., 2012 [19]	European sea bass, gilthead seabream, cod and salmon	SPME-GC/MS analysis of volatile compounds; statistical processing by principal component analysis and ascending hierarchical classification	dimethyl sulfide, 3-methylbutanal, ethyl acetate and 2-methylbutanal
Iglesias et al., 2009 [20]	Gilthead sea bream	SPME-GC/MS analysis of volatile compounds; peroxide value; thiobarbituric acid-reactive substances (TBARS) index	1-octen-3-ol, 1-penten-3-ol, and Z-4-heptenal
Metabolomic nuclear magnetic resonance spectroscopy (NMR) approach			
Shumilina et al., 2020 [21]	Salmon	NMR characterization; chemometrics statistical evaluation	Measurement of aspartate in frozen/thawed samples
Metabolomic high resolution mass spectrometry (HRMS) approach			
This study	Salmon, bullet tuna	HRMS characterization; multivariate data analysis	- Arginine and its metabolites - Anserine - Lysine and its metabolites - γ-glutamyl-S-methylcysteinyl-β-alanine - N-methyl-ethanolamine phosphate - l-α-Glyceryl-phosphoryl-choline
Combination of different analytical strategies			
Duflos et al., 2002 [22]	Plaice, whiting, mackerel	Physical determination (torrymeter); physiological examination (eye lens); enzymatic assays: (α-glucosidase, β-N-acetylglucosaminidase and β-hydroxyacyl-CoA-dehydrogenase)	- modification of the electrical properties of tissue by torrymeter - β-hydroxyacyl-CoA-dehydrogenase and α-glucosidase enzymatic assays
Popelka et al., 2014 [23]	Rainbow trout	Chemical composition (water, protein and fat content); expressible drip; total volatile nitrogen (TVN); microbiological analyses; histological examinations	- higher expressible drip and TVN in frozen samples - defects of the muscle structure of frozen fish
Marlard et al., 2019 [24]	Sea bass exudate	Proteins amount; α-d-glucosidase specific activity; nucleotides and related compounds (NRCs); free Ca^2+^	NRs and Ca^2+^ concentration

**Table 2:** The main differentiators detected in salmon samples as proposed markers for fresh and frozen discrimination (mean ± SD; µg/IS equivalents ^1^).

Compound	Fresh	Frozen/Thawed				*p* Values ^2^, Regulation ^3^
		−20 °C		−35 °C		
		Blast Chiller	Cold Room	Blast Chiller	Cold Room	
T0–hawed samples analyzed immediately						
Anserine (β-alanyl-3-metil l-histidine)	2214.1 ± 228.8	1197.3 ± 422.9	1312.3 ± 553.3	1521.2 ± 541.3	1638.2 ± 221.3	0.025, ↓
Arginine	24.3 ± 9.8	38.3 ± 6.4	39.7 ± 16.4	32.3 ± 7.9	35.8 ± 17.1	<0.001, ↑
Agmatine	12.0 ± 20.8	0.3 ± 0.5	0.4 ± 0.7	0.3 ± 0.3	0.1 ± 0.2	<0.001, ↓
Adma/Sdma (N, N-dimethyl-arginine)	4.3 ± 2.8	8.3 ± 2.1	7.3 ± 2.9	6.8 ± 1.8	10.0 ± 3.9	0.028, ↑
Lysine	35.5 ± 5.2	57.1 ± 23.8	52.3 ± 17.9	51.6 ± 13.4	63.2 ± 12.3	0.017, ↑
Pipecolic acid	1.7 ± 0.7	3.7 ± 2.7	5.2 ± 3.8	13.8 ± 8.1	25.8 ± 1.1	0.011, ↑
1-Piperideine	5.5 ± 2.1	9.6 ± 4.3	10.2 ± 3.4	10.3 ± 2.71	12.2 ± 1.9	<0.001, ↑
Phenylalanine	15.7 ± 7.3	25.9 ± 4.2	25.6 ± 4.0	22.9 ± 1.5	28.2 ± 8.4	0.008, ↑
Taurine	47.7 ± 7.1	25.9 ± 4.2	38.6 ± 4.0	24.9 ± 2.5	38.2 ± 0.4	0.006, ↓
T10–thawed samples analyzed after 10 days refrigerator (4 °C) storage						
Histidine	94.1 ± 41.1	124.7 ± 37.2	124.2 ± 43.2	130.6 ± 32.6	158.6 ± 64.6	0.005, ↑
Histamine	38.2 ± 13.3	1.6 ± 1.55	2.8 ± 1.8	1.9 ± 2.9	2.5 ± 2.6	<0.001, ↓
Arginine	11.4 ± 12.3	78.5 ± 9.2	113.47 ± 70.3	56.3 ± 17.9	48.8 ± 31.1	<0.001, ↑
Agmatine	124.8 ± 82.5	11.6 ± 16.1	13.1 ± 10.2	19.2 ± 0.3	20.2 ± 2.2	<0.001, ↓
Adma/Sdma (N, N-dimethyl-arginine)	5.2 ± 3.1	11.1 ± 2.0	19.6 ± 2.9	19.8 ± 7.7	20.0 ± 13.9	0.021, ↑
N-acyl-carnitine	34.8 ± 12.5	66.4 ± 13.2	55.1 ± 27.2	53.3 ± 11.2	62.2 ± 21.9	0.043, ↑
Lysine	22.8 ± 12.1	59.1 ± 26.8	93.3 ± 53.9	62.0 ± 26.4	97.4 ± 24.3	0.036, ↑
Cadaverine	27.8 ± 9.1	n.d.^4^	n.d.	n.d.	n.d.	<0.001, ↓
d-Pipecolic acid	12.7 ± 12.7	33.4 ± 2.7	35.2 ± 14.8	23.8 ± 11.1	45.8 ± 11.1	<0.001, ↑
1-Piperideine	5.7 ± 2.75	16.5 ± 4.70	19.45 ± 9.97	13.33 ± 5.86	20.5 ± 11.4	<0.001, ↑
Serine	1.6 ± 1.3	7.2 ± 1.1	7.5 ± 3.7	4.9 ± 2.3	7.4 ± 1.0	<0.001, ↑
Glutamic acid	11.0 ± 7.1	19.2 ± 2.5	22.0 ± 12.5	18.2 ± 7.4	22.9 ± 15.6	0.039, ↑
Pyroglutamic acid	4.5 + 1.5	9.9 ± 2.1	10.8 ± 4.2	8.2 ± 3.1	11.8 ± 5.2	0.006, ↑
Phenylalanine	3.6 ± 1.5	10.7 ± 1.8	12.1 ± 8.6	10.0 ± 3.6	18.2 ± 12.4	0.005, ↑
Tyrosine	23.6 ± 12.8	49.9 ± 7.2	57.6 ± 19.0	36.9 ± 13.5	53.2 ± 32.4	<0.001,↑
Taurine	8.1 ± 2.1	15.9 ± 4.1	13.6 ± 24.0	14.8 ± 1.5	18.2 ± 1.4	<0.001, ↑
N-methyl-ethanolamine phosphate	23.8 ± 12.9	32.67 ± 5.8	54.1 ± 2.5	39.82 ± 18.2	47.2 ± 12.8	<0.001, ↑
Trimethylamine N-oxide	264.6 ± 26.25	627.2 ± 123.2	590.0 ± 37.12	438.1 ± 123.2	541.0 ± 54.1	<0.001, ↑

^1^ µg/IS equivalents–µg/internal standard equivalents; ^2^ Statistical significance for fresh vs. frozen/thawed samples was evaluated by one-way ANOVA followed by Dunnett post hoc test; ^3^ ↑ the metabolites were up-regulated, ↓ the metabolites were down-regulated, ^4^ n.d.–not detected.

**Table 3:** The main down- and up-regulated metabolites identified as proposed markers for fresh and frozen discrimination in the bullet tuna sample (mean ± SD; µg/g SI equivalents ^1^).

Compound	Fresh	Frozen/Thawed			*p* Values ^2^, Regulation ^3^
		15 Days	30 Days	90 Days	
Arginine	22.4 ± 12.3	58.5 ± 19.2	43.7 ± 20.1	66.3 ± 7.9	0.0038, ↑
γ-glutamyl-S-methylcysteinyl-β-alanine	109.2 ± 12.8	12.4 ± 4.7	10.7 ± 5.3	7.1 ± 4.7	<0.001, *↓*
N-methyl-ethanolamine-phosphate	42.8 ± 22.5	100.6 ± 10.2	205.1 ± 98.5	350.8 ± 10.2	<0.001, ↑
l-α-Glyceryl-phosphoryl-choline	7.8 ± 7.6	52.2 ± 12.2	42.1 ± 12.8	20.7 ± 14.9	<0.001, ↑

^1^ µg/IS equivalents—µg/internal standard equivalents; ^2^ Statistical significance for fresh vs. frozen/thawed samples was evaluated by one-way ANOVA followed by Dunnett post hoc test; ^3^ ↑ the metabolites were up-regulated, ↓ the metabolites were down-regulated.

## Supplementary Materials

The following are available online at https://www.mdpi.com/2304-8158/9/12/1896/s1, Figure S1: Workflow tree from the Compound Discoverer 3.1 software displaying select data processing nodes and the associated workflow connections. Figure S2: PCA projection on the distribution of fresh and pulled frozen/thawed salmon samples as regards PC-1 with PC-2 when HRMS spectral characterisation of normalised peak area was used. Figure S3: The most important alterations in relative content (normalized peak aria) of amino acids and some of their metabolites in fresh salmon samples with corresponding preservation method. Table S1: Identification and semi-quantification of the metabolites detected in salmon samples at T0 (mean ± SD; µg/g IS equivalents). Table S2: Identification and semi-quantification of the metabolites detected in salmon samples at T10 (mean ± SD; µg/g IS equivalents) Table S3: Identification and semi-quantification of the metabolites detected in the Bullet tuna sample (mean ± SD; µg/g IS equivalents)
