# Supplementary material for: Discrimination between Fresh and Frozen-Thawed Fish Involved in Food Safety and Fraud Protection
Source: Foods. 2020 Dec 18;9(12):1896. doi: 10.3390/foods9121896 (PMC7766691; doi:10.3390/foods9121896)
Supplement: Supplementary file 1 [file foods-09-01896-s001.pdf]

SUPPLEMENTARY MATERIAL

Discrimination between fresh and frozen-thawed fish involved in food safety and fraud protection

Luca Maria Chiesa[a]. Radmila Pavlovic[a*]. Maria Nobile[a]. Federica di Cesare[a]. Renato Malandra[b]. Davide Pessina. Sara Panseri [a]

[a]Department of Health. Animal Science and Food Safety. University of Milan. Via Celoria 10. 20133 Milan. Italy

* Corresponding author:
Radmila Pavlovic: Via Celoria. 10 - 20133 Milan (MI) Italy
Tel: +390250317931 Fax: +390250317941
radmila.pavlovic1@unimi.it

Figure S1. Workflow tree from the Compound Discoverer 3.1 software displaying select data processing nodes and the associated workflow connections.

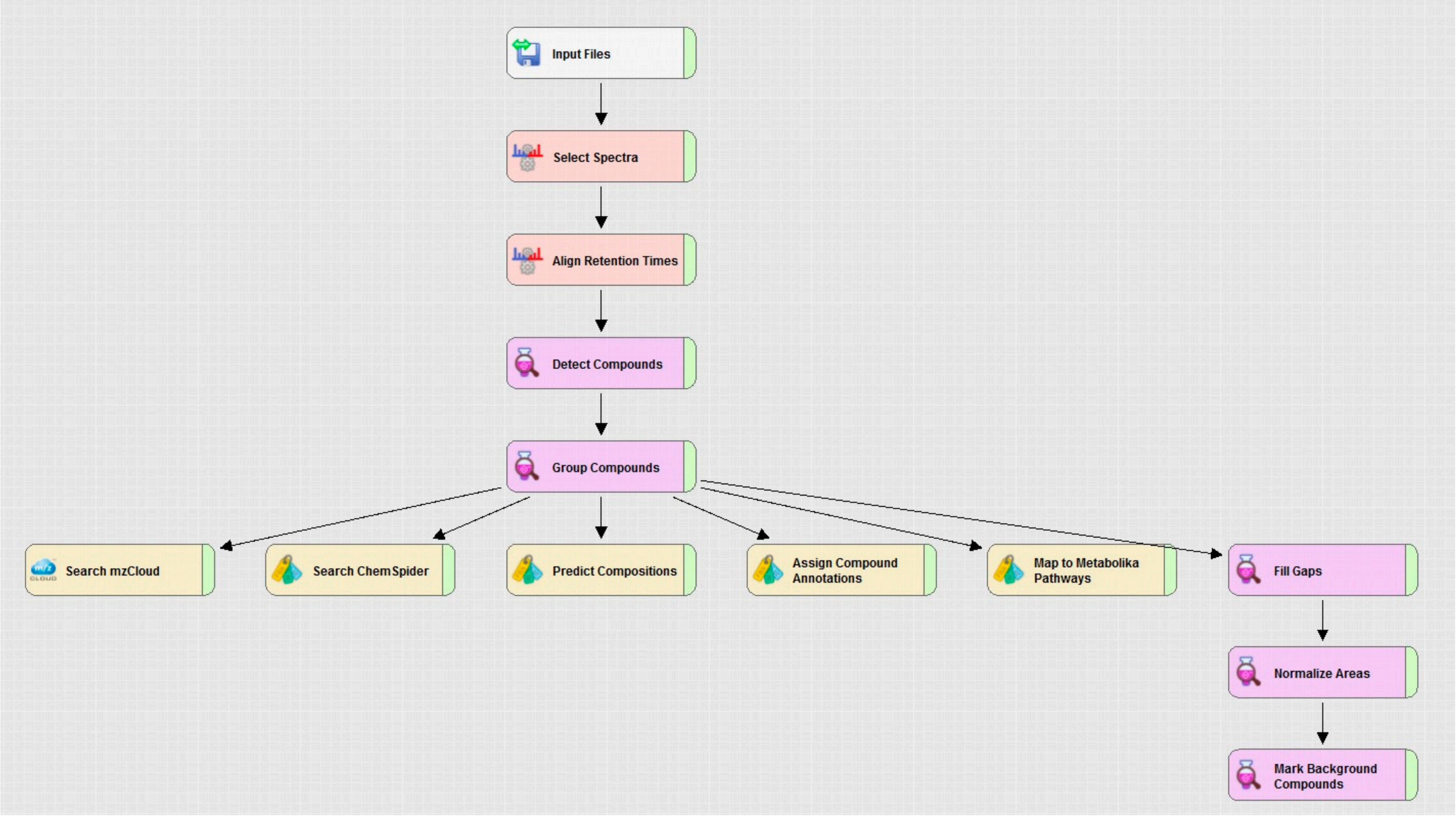

Figure S2. PCA projection on the distribution of fresh and pulled frozen/thawed salmon samples as regards PC-1 with PC-2 when HRMS spectral characterisation of normalised peak area was used.

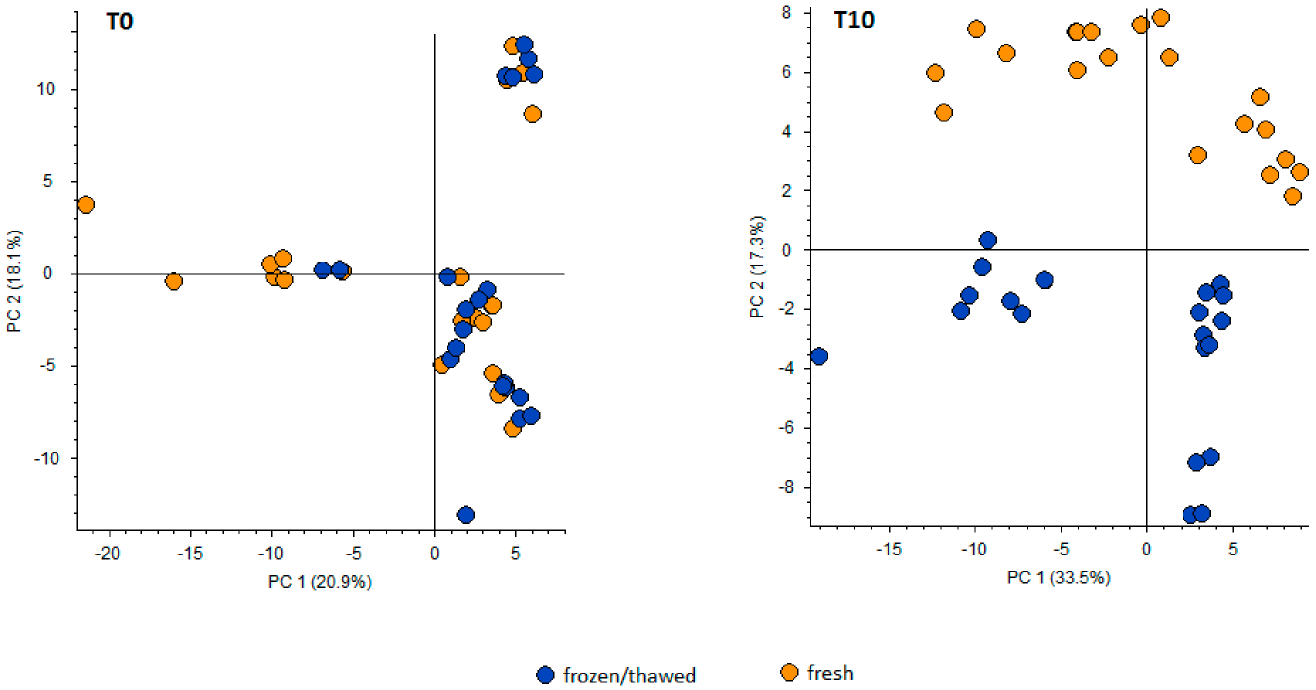

Figure S3. The most important alterations in relative content (normalized peak aria) of amino acids and some of their metabolites in fresh salmon samples with corresponding preservation method.

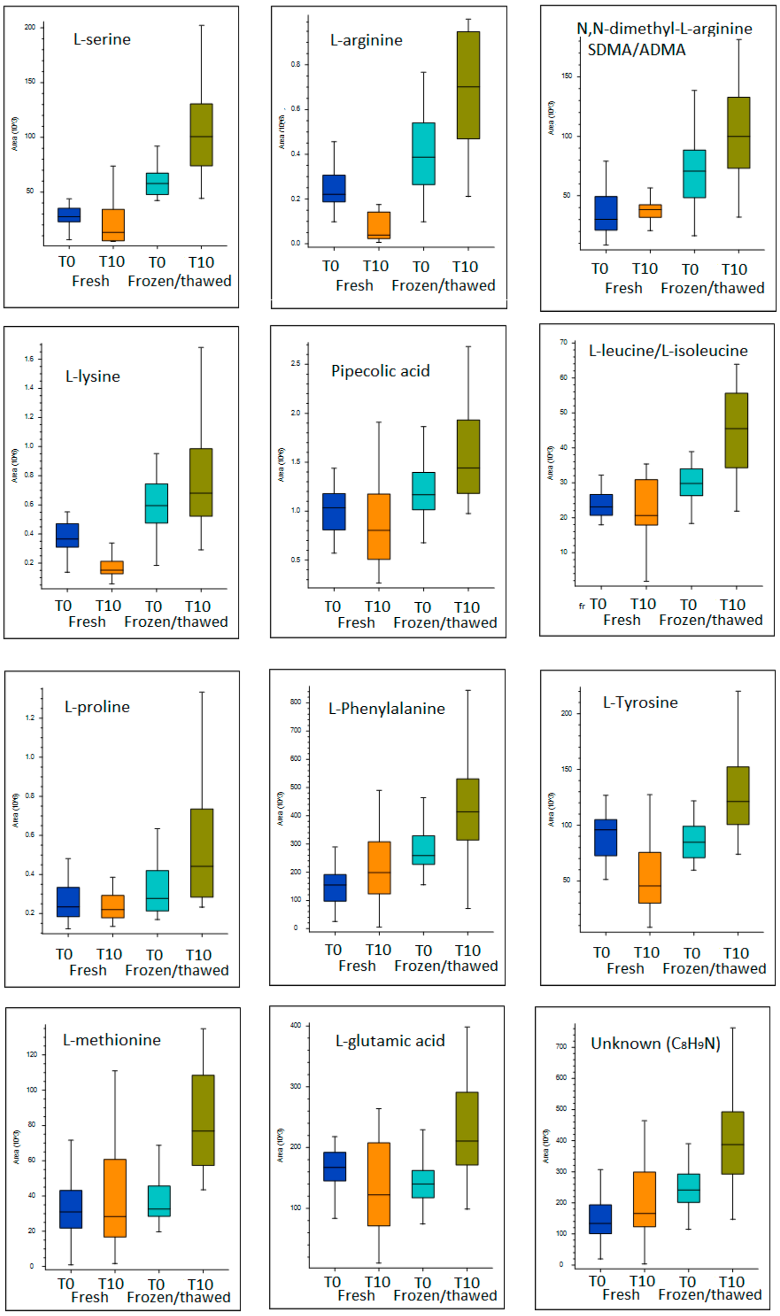

The box diagram representing the interval distribution between 25 and 75%. The horizontal line in the middle of the box represents the median.

Table S1. Identification and semi-quantification of the metabolites detected in salmon samples at T0 (mean ± SD; μg/g IS equivalents)

Compound	Formula	Exact Mass	Fresh	Frozen/ Thawed			
				- 20 °C		- 35 °C	
				blast chiller	cold room	blast chiller	cold room
Histidine and its metabolites/ derivatives							
Histidine	*C6H9N3O2*	*156.07675*	80.1 ± 41.1	92.6 ± 41.4	92.7 ± 51.7	82.4 ± 42.0	95.4 ± 55.4
Histamine	*C5H9N3*	*112.08692*	0.3 ± 0.2	0.6 ± 0.7	0.2 ± 0.2	0.2 ± 0.1	0.5 ± 0.6
3-Methyl-histidine	*C7H11N3O2*	*170.09240*	468.1 ± 105.1	392.3 ± 81.6	396.8 ± 82.7	326.0 ± 109.6	338.2 ± 74.3
3-Methyl-histamine	*C6H11N3*	*126.10257*	34.3 ± 7.6	28.0 ± 5.6	29.1 ± 5.7	32.3 ± 12.8	34.8 ± 15.7
Anserine (β-alanyl-3-metil L-histidine)	*C10H16N4O3*	*241.12950*	2214.1 ± 228.8	1197.3 ± 422.9	1312.3 ± 553.3	1521.2 ± 541.3	1638.2 ± 221.3
Carnosine (β-alanyl-l-histidine)	*C9H14N4O3*	*227.11386*	0.7 ± 0.2	0.4 ± 0.3	0.5 ± 0.4	0.2 ± 0.1	0.2 ± 0.1
Arginine and its metabolites/ derivatives							
Arginine	*C6H14N4O2*	*175.119*	24.3 ± 9.8	38.3 ± 6.4	39.7 ± 16.4	32.3 ± 7.9	35.8 ± 17.1
Agmatine	*C5H14N4*	*131.1291*	12.0 ± 20.8	0.3 ± 0.5	0.4 ± 0.7	0.3 ± 0.3	0.1 ± 0.2
Adma/Sdma (dimethyl-arginine)	*C8H18N4O2*	*203.1503*	4.3 ± 2.8	8.3 ± 2.1	7.3 ± 2.9	6.8 ± 1.8	10.0 ± 3.9
Nma (metil arginine)	*C7H16N4O2*	*189.1346*	0.1 ± 0.5	0.5 ± 0.3	1.2 ± 0.8	0.9 ± 0.2	1.6 ± 0.6
Ornithine	*C5H12N2O2*	*133.0972*	4.8 ± 6.5	5.3 ± 6.3	8.1 ± 5.0	6.7 ± 5.5	6.6 ± 5.6
Carnitine	*C7H15NO3*	*162.1125*	48.8 ± 13.2	42.0 ± 20.1	40.8 ± 17.5	25.5 ± 5.5	36.5 ± 7.0
N-acyl-carnitine	*C9H17NO4*	*204.1230*	56.1 ± 23.0	43.2 ± 12.4	52.4 ± 14.4	48.5 ± 19.6	53.9 ± 16.7
Spermine	*C12H28N4O*	*245.2336*	24.9 ± 6.9	18.0 ± 5.9	26.5 ± 9.7	15.8 ± 2.9	28.0 ± 10.1
Spermidine	*C7H19N3*	*146.1652*	36.9 ± 16.6	34.8 ± 5.3	27.3 ± 11.9	30.5 ± 12.4	32.8 ± 16.7
Creatinine	*C4H7N3O*	*114.0661*	31.9 ± 8.8	28.8 ± 1.2	26.7 ± 20.0	26.1 ± 6.9	27.2 ± 2.8
Creatine	*C4H4N3O2*	*132.0767*	3199.5 ± 598.6	2896.9 ± 521.2	3230.7 ± 940.2	2171.2 ± 677.2	2907.1 ± 189.6
Lysine and its metabolites/ derivatives							
Lysine	*C6H14N2O2*	*147.1128*	35.5 ± 5.2	57.1 ± 23.8	52.3 ± 17.9	51.6 ± 13.4	63.2 ± 12.3
Cadaverine	*C5H14N2*	*103.1230*	nd*	nd	nd	nd	nd
D-Pipecolic acid	*C6H11NO2*	*130.0863*	2.7 ± 0.7	3.7 ± 2.7	5.2 ± 3.8	13.8 ± 8.1	25.8 ± 1.1
1-Piperideine	*C5H9N*	*84.0808*	7.5 ± 2.1	9.6 ± 4.3	10.2 ± 3.4	10.3 ± 2.1	12.2 ± 1.9
Other amino acids and their metabolites/derivatives							
Proline	*C5H9NO2*	*116.0706*	42.5 ± 8.4	29.2 ± 13.9	48.1 ± 22.0	27.9 ± 9.1	24.0 ± 11.3
Leucine/isoleucine	*C6H13NO2*	*132.1019*	27.6 ± 9.3	29.2 ± 13.9	45.1 ± 18.0	27.9 ± 9.1	34.0 ± 21.3
Alanine	*C3H7NO2*	*90.0550*	72.5 ± 12.4	65.9 ± 9.9	76.1 ± 19.8	64.5 ± 15.9	67.7 ± 6.4
Serine	*C3H7NO3*	*106.0499*	2.0 ± 0.9	3.2 ± 1.1	3.0 ± 0.7	2.5 ± 0.3	3.4 ± 1.0
Valine	*C5H11NO2*	*118.0863*	149.7 ± 76.5	130.7 ± 20.1	201.7 ± 163.2	100.7 ± 24.9	161.2 ± 51.4
Glutamic acid	*C5H9NO4*	*148.0604*	14.4 ± 4.2	11.2 ± 2.2	11.0 ± 2.4	9.8 ± 3.6	8.9 ± 1.6
Pyroglutamic acid	*C5H7NO3*	*130.0499*	4.81 ± 1.62	8.01 ± 7.85	14.06 ± 16.25	3.92 ± 1.16	4.0 ± 1.3
Methionine	*C5H11NO2S*	*150.0583*	5.4 ± 3.1	4.5 ± 0.8	6.3 ± 2.6	5.0 ± 2.0	5.2 ± 2.4
Phenylalanine	*C9H11NO2*	*166.0863*	15.7 ± 7.3	25.9 ± 4.2	25.6 ± 4.0	22.9 ± 1.5	28.2 ± 8.4
Tyrosine	*C9H11NO3*	*182.0812*	12.2 ± 5.0	10.8 ± 1.9	12.2 ± 4.3	10.4 ± 3.9	11.4 ± 1.6

Tryptophan	*C11H12N2O2*	*205.0972*	2.2 ± 0.6	2.0 ± 0.3	1.8 ± 0.3	1.7 ± 0.1	1.9 ± 0.8
Cysteine	*C6H12N2O4S2*	*241.0311*	3.0 ± 0.8	2.2 ± 0.8	2.6 ± 1.0	1.9 ± 0.8	2.1 ± 0.5
Taurine	*C2H7NO3*	*126.0219*	17.7 ± 7.1	25.9 ± 4.2	38.6 ± 4.0	24.9 ± 2.5	38.2 ± 0.4
O-phosphate amino derivate							
N-methyl-ethanolamine phosphate	*C3H10NO4P*	*156.0420*	27.78 ± 13.01	32.4 ± 22.2	35.65 ± 20.92	16.58 ± 3.60	30.4 ± 3.1
Amino oxides							
Trimethylamine N-oxide	*C3H9NO*	*76.0757*	581.24 ± 217.67	508.25 ± 201.10	543.99 ± 226.2	535.84 ± 119.04	664.5 ± 195.25
Purines base							
Hypoxanthine	*C5H4N4O*	*137.0458*	264.6 ± 51.6	222.9 ± 57.2	175.2 ± 112.3	211.2 ± 51.4	239.2 ± 0.5
Xanthine	*C5H4N4O2*	*153.0407*	0.3 ± 0.1	0.2 ± 0.07	0.1 ± 0.2	0.1 ± 0.1	0.1 ± 0.1

*nd-not detected

Table S2. Identification and semi-quantification of the metabolites detected in salmon samples at T10 (mean ± SD; µg/g IS equivalents)

Compound	Formula	Exact Mass	Fresh	Frozen/ Thawed			
				- 20 °C		- 35 °C	
				blast chiller	cold room	blast chiller	cold room
Histidine and its metabolites/ derivatives							
Histidine	*C5H9N3O2*	*156.07675*	94.1 ± 41.1	124.7 ± 37.2	124.2 ± 43.2	130.6 ± 32.6	158.6 ± 64.6
Histamine	*C6H9N3*	*112.08692*	38.2 ± 13.3	1.6 ± 1.55	2.8 ± 1.8	1.9 ±2.9	2.5 ± 2.6
3-Methyl-histidine	*C7H11N3O2*	*170.09240*	551.2 ± 222.7	566.9 ±96.8	547.7 ± 190.2	476.9 ± 145.9	515.3 ± 189.9
3-Methyl-histamine	*C6H11N3*	*126.10257*	42.2 ± 10.2	40.0 ± 6.8	40.0 ± 11.9	35.3 ± 11.3	36.3 ± 11.2
Anserine (β-alanyl-3-metil L-histidine)	*C10H16N4O3*	*241.12950*	2362.2 ± 668.3	2210.9 ± 646.1	2297.9 ± 622.1	2122.4 ± 651.9	1910.4 ± 493.4
Carnosine (β-alanyl-l-histidine)	*C9H14N4O3*	*227.11386*	0.8 ± 0.7	1.0 ± 0.7	1.6 ± 1.1	0.6 ± 0.5	0.6 ± 0.4
Arginine and its metabolites/ derivatives							
Arginine	*C6H14N4O2*	*175.119*	11.4 ± 12.3	78.5 ± 9.2	113.4.7 ±70.3	56.3 ± 17.9	48.8 ± 31.1
Agmatine	*C5H14N4*	*131.1291*	124.8 ± 82.5	11.6 ± 16.1	13.1 ± 10.2	19.2 ± 0.3	20.2 ± 2.2
Adma/Sdma (dimethyl-arginine)	*C8H18N4O2*	*203.1503*	5.2 ± 3.1	11.1 ± 2.0	19.6 ± 2.9	19.8 ± 7.7	20.0 ± 13.9
Nma (methyl-arginine)	*C7H16N4O2*	*189.1346*	0.7 ± 0.5	8.5 ± 11.4	1.8 ± 0.2	0.6 ± 0.3	1.7 ± 1.6
Ornithine	*C5H12N2O2*	*133.0972*	8.9 ± 6.8	8.2 ± 2.0	12.9 ± 10.0	1.7 ± 0.8	6.1 ± 5.5
Carnitine	*C7H15NO3*	*162.1125*	76.8 ± 41.7	61.5 ± 12.3	60.2 ± 12.2	46.8 ± 17.7	49.8 ± 22.4
N-acyl-carnitine	*C9H17NO4*	*204.1230*	34.8 ± 12.5	66.4 ± 13.2	55.1 ± 27.2	53.3 ± 11.2	62.2 ± 21.9
Spermine	*C12H28N4O*	*245.2336*	38.6 ±10.4	36.0 ± 1.9	33.5 ± 1.7	32.8 ± 12.9	28.0 ± 10.1
Spermidine	*C7H19N3*	*146.1652*	37.9 ± 267	44.8 ± 10.3	17.3 ± 10.9	31.5 ± 7.4	29.8 ± 15.7
Creatinine	*C4H7N3O*	*114.0661*	38.4 ± 8.2	34.8 ± 1.3	33.6 ± 3.8	30.1 ± 7.0	48.2 ± 22.8
Creatine	*C4H4N3O2*	*132.0767*	3286.2 ± 1125.2	3269.2.9 ± 328.2	3588.7 ± 576.2	3371.2 ± 642.2	3319.1 ± 914.6
Lysine and its metabolites/ derivatives							
Lysine	*C6H14N2O2*	*147.1128*	22.8 ± 12.1	59.1 ± 26.8	93.3 ± 53.9	62.0 ± 26+.4	97.4 ± 24.3
Cadaverine	*C5H14N2*	*103.1230*	27.8 ± 9.1	nd*	nd	nd	nd
D-Pipecolic acid	*C6H11NO2*	*130.0863*	12.7 ± 12.7	33.4 ± 2.7	35.2 ± 14.8	23.8 ± 11.1	45.8 ±1 1.1
1-Piperideine	*C5H9N*	*84.0808*	5.7 ± 2.75	16.5 ± 4.70	19.45 ± 9.97	13.33 ± 5.86	20.5 ± 11.4
Other amino acids and their metabolites/derivatives							
Alanine	*C3H7NO2*	*90.0550*	72.5 ± 12.4	65.9 ± 9.9	76.1 ± 19.8	64.5 ± 15.9	67.7 ± 6.4
Serine	*C3H7NO3*	*106.0499*	1.6 ± 1.3	7.2 ± 1.1	7.5 ± 3.7	4.9 ± 2.3	7.4 ± 1.0
Valine	*C5H11NO2*	*118.0863*	180.1 ± 99.4	180.7 ± 7.7	372.3 ± 21.7	200.6 ± 106.9	310.2 ± 106.4
Glutamic acid	*C5H9NO4*	*148.0604*	11.0 ±7.1	19.2 ± 2.5	22.0 ± 12.5	18.2 ± 7.4	22.9 ± 15.6
Pyroglutamic acid	*C5H7NO3*	*130.0499*	4.5 + 1.5	9.9 ± 2.1	10.8 + ±4.2	8.2 ± 3.1	11.8 ±5.2
Methionine	*C5H11NO2S*	*150.0583*	5.4 ± 3.1	4.5 ± 0.8	6.3 ± 2.6	5.0 ± 2.0	5.2 ± 2.4
Phenylalanine	*C9H11NO2*	*166.0863*	3.6 ± 1.5	10.7 ± 1.8	12.1 ± 8.6	10.0 ± 3.6	18.2 ± 12.4
Tyrosine	*C9H11NO3*	*182.0812*	23.6 ± 12.8	49.9 ± 7.2	57.6 ± 19.0	36.9 ± 13.5	53.2 ± 32.4
Tryptophan	*C11H12N2O2*	*205.0972*	2.8 ± 1.2	4.0 ± 0.4	4.8 ± 2.5	3.0 ± 0.1	5.3 ± 5.1
Cysteine	*C6H12N2O4S2*	*241.0311*	3.8 ± 1.4	3.4 ±1.7	3.2 ± 1.3	3.1 ± 1.7	2.2 ± 0.5
Taurine	*C2H7NO3*	*126.0219*	8.1 ±2.1	15.9 ± 4.1	13.6 ±24.0	14.8 ± 1.5	18.2 ± 1.4

O-phosphate amino derivate							
N-methyl-ethanolamine phosphate	*C3H10NO4P*	*156.0420*	23.8 ± 12.9	32.67 ± 5.8	54.1 ± 2.5	39.82 ± 18.2	47.2 ± 12.8
Amino oxides							
Trimethylamine N-oxide	*C3H9NO*	*76.0757*	264.6 ± 26.25	627.2 ± 123.2	590.0 ± 37.12	438.1 ± 123.2	541.0 ± 54.1
Purines base							
Hypoxanthine	*C5H4N4O*	*137.0458*	400.9 ± 104.0	365.2 ± 53.2	350.6 ± 90.3	290.2 ± 95.4	317.2 ± 134.5
Xanthine	*C5H4N4O2*	*153.0407*	2.1 ± 1.8	0.5 ± 0.3	0.3 ± 0.1	0.2 ± 0.1	0.4 ± 0.1

*nd-not detected

Table S3. Identification and semi-quantification of the metabolites detected in the Bullet tuna sample (mean ± SD; μg/g IS equivalents)

	Formula	Exact Mass	Fresh	Frozen/ Thawed		
				15days	30days	90days
Histidine and its metabolites/ derivatives						
Histidine	*C5H9N3O2*	*156.07675*	8294.1 ± 278.1	1024.8 ± 125.2	1024.2 ± 43.2	1300.6 ± 32.6
Histamine	*C6H9N3*	*112.08692*	nd	nd	0.9 ± 0.5	nd
3-Methyl-histidine	*C7H11N3O2*	*170.09240*	1022.2 ± 227.7	22.9 ±16.8	57.7 ± 10.2	86.9 ± 45.8
3-Methyl-histamine	*C6H11N3*	*126.10257*	22.7 ± 12.7	33.4 ± 2.7	35.2 ± 14.8	23.8 ± 11.1
Anserine (β-alanyl-3-metil L-histidine)	*C10H16N4O3*	*241.12950*	82.1 ± 8.3	6.9 ± 1.1	5.9 ± 32.1	4.4 ± 2.9
Carnosine (β-alanyl-l-histidine)	*C9H14N4O3*	*227.11386*	9.4 ± 1.2	nd	nd	nd
Arginine and its metabolites/ derivatives						
Arginine	*C6H14N4O2*	*175.119*	22.4 ±12.3	58.5 ± 19.2	43.7 ±20.1	66.3 ± 7.9
Agmatine	*C5H14N4*	*131.1291*	2.8 ±0.3	nd	nd	nd
Adma/Sdma -dimethyl-arginine)	*C8H18N4O2*	*203.1503*	0.4 ±0.1	nd	nd	nd
Nma (methyl- arginine)	*C7H16N4O2*	*189.1346*	0.9 ±0.3	nd	nd	nd
Ornithine	*C5H12N2O2*	*133.0972*	4.4 ±42.3	nd	nd	nd
Carnitine	*C7H15NO3*	*162.1125*	146.8 ± 21.7	162.5 ± 48.3	160.2 ± 22.2	126.8 ± 27.7
N-acyl-carnitine	*C9H17NO4*	*204.1230*	44.8 ± 12.5	66.4 ± 33.2	58.1 ± 27.2	63.3 ± 21.2
Spermine	*C12H28N4O*	*245.2336*	64.2 ±70.4	62.0 ± 11.9	53.5 ± 10.7	42.8 ± 22.9
Spermidine	*C7H19N3*	*146.1652*	27.9 ± 16.7	24.8 ± 19.3	37.3 ± 15.9	41.5 ± 17.4
Creatinine	*C4H7N3O*	*114.0661*	80.4 ± 40.2	14.7 ± 9.7	7.1 ± 45.9	4.5 ± 7.4
Creatine	*C4H4N3O2*	*132.0767*	6258.2 ± 925.2	3125.9 ± 415.2	3222.7 ± 586.2	3371.2 ± 1652.2
Lysine and its metabolites/ derivatives						
Lysine	*C6H14N2O2*	*147.1128*	122.8 ± 42.1	109.1 ± 16.8	83.3 ± 13.9	72.0 ± 46+.4
Cadaverine	*C5H14N2*	*103.1230*	nd	nd	nd	nd
Other amino acids and their metabolites/derivatives						
Proline	*C5H9NO2*	*116.0706*	102.2 ± 2.3	30.4 ±11.7	30.2 ± 15.3	30.1 ± 10.7
Alanine	*C3H7NO2*	*90.0550*	122.5 ± 12.4	65.9 ± 9.9	66.1 ± 18.8	44.5 ± 11.9
Valine	*C5H11NO2*	*118.0863*	2521.1 ± 129.4	100.7 ± 7.7	72.0 ± 8.7	125.6 ± 84.9
Glutamic acid	*C5H9NO4*	*148.0604*	41.0 ± 9.1	8.2 ± 3.5	12.0 ± 2.5	7.2 ± 2.4
Pyroglutamic acid	*C5H7NO3*	*130.0499*	28.5 + 12.5	8.9 ± 1.1	7.8 + ±1.2	1.2 ± 3.1
Methionine	*C5H11NO2S*	*150.0583*	15.4 ± 8.1	4.5 ± 0.8	5.3 ± 2.6	1.0 ± 0.8
Phenylalanine	*C9H11NO2*	*166.0863*	96.6 ± 14.5	20.7 ± 2.8	22.1 ± 8.6	10.0 ± 3.6
Tyrosine	*C9H11NO3*	*182.0812*	1.8 ± 1.4	nd	nd	nd
Tryptophan	*C11H12N2O2*	*205.0972*	42.8 ± 21.2	2.0 ± 0.5	1.8 ± 0.5	5.1 ± 0.8
Cysteine	*C6H12N2O4S2*	*241.0311*	13.8 ± 11.4	4.4 ±1.8	3.1 ± 0.3	2.1 ± 0.7
Taurine	*C2H7NO3*	*126.0219*	18.1 ± 3.1	0.8 ± 0.1	3.6 ±2.0	2.8 ± 0.5
peptides						
γ-glutamyl-S-methylcysteinyl-β-alanine	*C12H21N3O6S*	*336.1225*	109.2 ± 12.8	12.4 ±4.7	10.7 ± 5.3	7.1 ± 4.7
Glutathione (Reduced)	*C10H17N3O6S*	*308.0908*	2.8 ± 1.2	nd	nd	nd
O-phosphate amino derivate						

N-methyl-ethanolamine phosphate	*C3H10NO4P*	*156.0420*	42.8 ± 22.5	100.6 ± 10.2	205.1 ± 98.5	350.8 ± 10.2
L-α-Glyceryl-phosphoryl-choline	*C3H10NO4P*	*258.1099*	7.8 ± 7.6	52.2 ± 12.2	42.1 ± 12.8	20.7 ± 14.9
Amino oxides						
Trimethylamine N-oxide	*C3H9NO*	*76.0757*	351.6 ± 226.25	225.2 ± 175.2	125.0 ± 37.12	447.1 ± 151.2
Purines base						
Hypoxanthine	*C5H4N4O*	*137.0458*	1320.9 ± 221.0	105.2 ± 13.2	125.6 ± 40.3	190.4 ± 85.4
Inosinic acid	*C10H13N4O8P*	*153.0407*	8.1 ± 2.2	1.1 ± 2.2	0.2 ± 0.1	2.8 ± 1.2

*nd-not detected
